# Daily Physical Activity and Sedentary Time Assessed by Acceleration Based on Mean Amplitude Deviation among Older People

**DOI:** 10.3390/ijerph17186887

**Published:** 2020-09-21

**Authors:** Ying Gao, Timo Rantalainen, Taija Finni, Erja Portegijs, Johanna Eronen, Taina Rantanen, Merja Rantakokko

**Affiliations:** 1 Department of Sports Science, College of Education, Zhejiang University, Hangzhou 310058, China; 2 Faculty of Sport and Health Sciences, University of Jyväskylä, 40014 Jyväskylä, Finland; timo.rantalainen@jyu.fi (T.R.); taija.m.juutinen@jyu.fi (T.F.); erja.portegijs@jyu.fi (E.P.); johanna.eronen@jyu.fi (J.E.); taina.rantanen@jyu.fi (T.R.); 3 School of Health and Social Studies, JAMK University of Applied Sciences, 40101 Jyväskylä, Finland; merja.rantakokko@jamk.fi

**Keywords:** mobility, inactivity, health promotion

## Abstract

Accelerometer-derived estimates of physical activity (PA) and sedentary time have been an important methodological focus. However, little is known about the daily activities among older people during their normal lives. Furthermore, some older individuals would like to be more active, yet experience an unmet PA need, which is defined as the desire to engage in more PA but without the opportunity to act on the desire. This study examined the intensity of daily PA and sedentary behavior measured with accelerometers among older people, and whether PA differs between weekdays and weekends and those with and without the experience of unmet PA need, measured with self-reports. A total of 174 community-dwelling older people (64% female) aged 75 to 90 years used an accelerometer for 7 consecutive days during waking hours, and the results were classified for sedentary behavior (thresholds of 0.0167 g), light activity (0.091 g), and moderate-to-vigorous activity (MVPA, 0.414 g) based on mean amplitude deviation (g). We found that during weekdays, older people engaged slightly more in light activity and had less sedentary time than during weekends. In total, 7.6% of the participants perceived an unmet PA need. Accordingly, those with unmet PA needs spent less time in MVPA, especially during weekdays, and they might benefit from PA-enabling interventions.

## 1. Introduction 

Engaging in moderate-to-vigorous physical activity (MVPA) is recommended to improve health among older people [1]. However, older people spend most of their awake time in a sitting or lying position as sedentary behavior [2]. With such high exposure to sedentary behaviors, the impact of it may be, independent of physical activity (PA), related to increased risks for mortality and morbidity [3,4]. Therefore, it has been suggested that an efficacious intervention for health promotion should likely target both a reduction in sedentary time and an increase in PA [5,6,7]. In order to enable the design of effective strategies and to identify opportunities for reduced sedentariness and increased PA among older people, further information regarding typical patterns of sedentary and active behaviors are needed. 

Some older people would prefer to increase their PA level, but at the same time feel that they lack the opportunity to do so [8]. We have defined such a situation as an unmet need for PA [8]. In other words, unmet PA need is a personal feeling that one’s PA level is inadequate [8], and thus is distinct from the concept of physical inactivity or guidelines for the sufficient amount of PA recommended for older people [9]. Our previous studies have shown that unmet PA need is more common among those with health problems, such as musculoskeletal diseases, mobility limitations, depressive symptoms and daytime tiredness, yet perceived also among those with good health [8,10,11]. Underlying causes of unmet PA need are not widely examined, but it is suggested that a reduction in physical activity level [12], maladaptive walking modifications, namely reduced frequency of walking or giving up walking long distances [13], lower socioeconomic status [10] and mobility limitations [14] increase the risk for development of the perception of unmet PA need. Associations with self-reported unmet PA need and the actual intensity of PA measured with accelerometers are not well known. When we assess the daily activities of older people, their perception of unmet PA need should be considered as an important factor that may provide possibilities for interventions targeted toward increasing PA and reducing sedentary time. 

The majority of studies have focused on the total volume of PA and sedentary time measured by accelerometers [15,16]. However, few studies reported the intra-individual variability of PA and sedentary time across days, and how PA patterns and sedentary behavior are accumulated within a day [17,18,19]. It is foreseeable that day-to-day variability exists, driven by the type of the day (e.g., weekday, weekend, holiday) and various concomitant factors (shopping, hobbies etc.). Some studies found that older adults promote or attenuate their total amount of activity on day-to-day variability [17,18,20], which may be related to social routines requiring mobility. These considerable determinants can be related to opportunities for promoting more PA and less sedentary time for older people. However, exploration of the topic remains relatively scarce. 

Therefore, this study aimed to examine the daily PA and sedentary time among older people and whether PA differs between weekdays and weekends and those with and without perception of unmet PA need.

## 2. Methods

### 2.1. Participants 

This study used the baseline data from the sub-study of LISPE project [21]. Details of the LISPE project and sub-study have been reported in detail previously [21]. Briefly, LISPE was a prospective cohort study focusing on individual and environmental determinants of life-space mobility among community-dwelling people. To be included in the study, participants needed to be aged 75 to 90 years, live independently in their own homes, and live in the study area in the cities of Jyväskylä and Muurame, Finland (*n* = 848). All participants were interviewed by phone and then face-to-face in their homes. During a three-month period from March 26th to June 15th, participants were offered the possibility to take part in a physical activity surveillance study by wearing an accelerometer and completing a 7-day activity diary (*n* = 174). In the final sample, 67% of the participants had no mobility limitations at all, 31% at most had minor difficulties, and only 2% had major difficulties, and they had a median of 4 (IQR = 4) chronic conditions [22]. The LISPE project was approved by the ethics committee of the University of Jyväskylä. All participants were informed of the procedures and they signed an informed consent form before any measurement. 

### 2.2. Participant Measures

Participant demographic characteristics were collected in a face-to-face interview by standardized protocols [21] They were instructed to wear an accelerometer (Hookie, tri-axial, “AM20 Activity Meter”, Hookie Technologies Ltd., Espoo, Finland) on a belt on their right hip for seven consecutive days during waking hours, except during water-related activities (e.g., bathing, taking a sauna, or swimming). Detailed instructions for the accelerometer were also given in writing. Participants were encouraged to maintain their usual daily routines during the measurement week. They were given an activity diary in which they recorded the dates and times at which they put on and took off the accelerometer during the seven days. 

### 2.3. Accelerometer Data Processing

The accelerometer measures accelerations in three individual x-, y-, and z- axes for vertical, horizontal, and perpendicular, and has a dynamic range of ±16 g, 13 bit at 100 Hz. The resultant acceleration of each sample was calculated and used in all further analyses. The mean amplitude deviation (MAD) [23,24] was calculated in non-overlapping 5 s epochs, which were subsequently averaged in one-minute epochs, using a custom-written Matlab script (R2015b, Mathworks, Inc., Natick, MA, USA). This process resulted in 1440 values per day (=24 h × 60 min/h * 1 min/epoch * 1 value/epoch). The pre-processed one-minute data were divided into 24 h segments from midnight to midnight, and further processing was done in 24 h segments. Non-wear time was defined as any continuous epochs of at least one hour with all of the one-minute MADs less than 0.024 g. This non-wear algorithm produced congruent results with self-reported accelerometer non-wear time. Any days with less than 10 h of wear-time and days with abnormal routines (e.g., sick at home or travelling) were excluded from the analysis.

Sedentary time and physical activity were assessed from each of the 24 h segments of one-minute MAD epoch values. The one-minute values were classified into sedentary (< 0.0167 g), light PA (0.0167 to < 0.091 g), moderate PA (0.091 to < 0.414 g), or vigorous PA (≥ 0.091 g) after excluding all non-wear minutes. The intensity cut-offs were based on the optimal classification for light PA [24], and at MADs corresponding to 3 metabolic equivalents, and 6 METs for moderate PA and vigorous PA, respectively [25]. The MVPA was summed from the time spent in moderate PA and vigorous PA. The mean minutes per day spent in sedentary, light, and MVPA were reported and the proportion of respective activity intensities per day was calculated based on daily recorded time. Sedentary bout and light and MVPA bout accumulation were further assessed based on the one-minute data from all recorded days [26] and all bouts lasting >10 min were identified [27]. Furthermore, each of the 24 h segments of one-minute MAD epoch values were averaged accordingly from Monday to Friday for weekdays and from Saturday to Sunday for weekends.

### 2.4. Unmet Physical Activity Need

Unmet PA need is the self-reported feeling that one’s level of PA is inadequate [8]. Unmet PA need was evaluated by the questions “Do you feel that you would have the opportunity to increase your level of physical activity if someone recommended you to do so?” and “Would you like to increase your level of physical activity?” The response options were yes and no. Participants who perceived no opportunity to increase their physical activity, but were willing to do so, were defined as experiencing an unmet PA need [8]. 

### 2.5. Statistical Analyses

Participant characteristics were described using means and standard deviations and their 95% confidence intervals (CI) or numbers with percentages. The total recorded time per day, the number of bouts per day, the mean minute of PA and sedentary time per day and the daily proportion of time spent in sedentary, light, and MVPA are reported in the results, and further averaged for weekdays (Monday to Friday) and weekends (Saturday and Sunday). The differences between weekdays and weekends and those with and without the perception of unmet PA need were analyzed using t-tests or non-parametric t-tests. For day-to-day variability, standard deviations were reported for accelerometer-measured sedentary time, light and MVPA. Statistical analyses were conducted using IBM SPSS for Windows Version 24.0 (IBM Corp., Armonk, NY, USA). A probability level of *p* < 0.05 (two-tailed) was considered statistically significant. 

## 3. Results

A total of 174 community-dwelling older people (64% female) aged 75 to 90 were included in this study, and of them, 7.6% participants (*n* = 13) perceived an unmet PA need. For accelerometer measurements, there were a total of 1103 valid days with at least a 10 h of recording time for all participants, which included 792 weekdays and 311 weekend days. Participants’ characteristics, PA, and sedentary time in minutes are reported in Table 1. 

**Figure 1:**
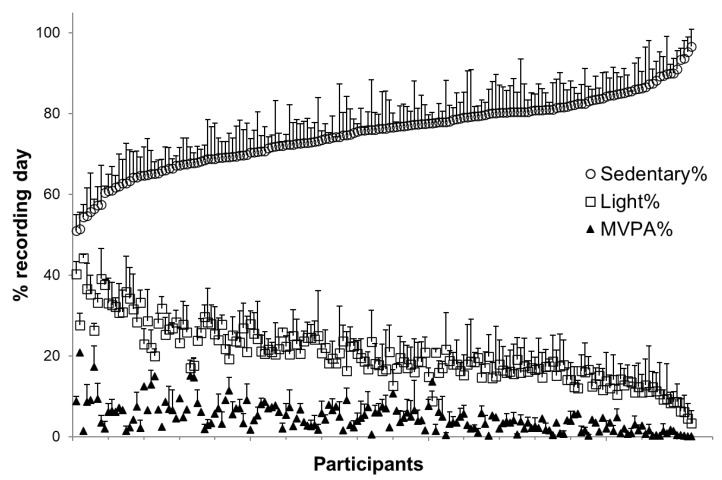
Individual time spend of sedentary, light physical activity (PA), and moderate-to-vigorous physical activity (MVPA). Data are organized according to the amount of measured sedentary time. Standard deviations denote day-to-day variation during the measurement week for each intensity activity. The x axis is labelled according to each participant.

The length of recorded time for all participants averaged 13.5 ± 1.3 h/d, consisting of 75.3 ± 8.7% (95% CI: 73.9–76.6%) sedentary time, 20.1 ± 7.2% (95%CI: 19.0–21.2%) light PA and 4.7 ± 3.5% (95%CI: 4.1–5.2%) MVPA. There were 16.6 ± 2.9 sedentary and 0.8 ± 0.9 light and MVPA bouts lasting >10 min/d (Table 1). The averaged individual day-to-day variability was 5.4 ± 2.6% for sedentary time, 4.7 ± 2.4% for light, and 2.4 ± 1.7% for MVPA (Figure 1).

**Figure 2:**
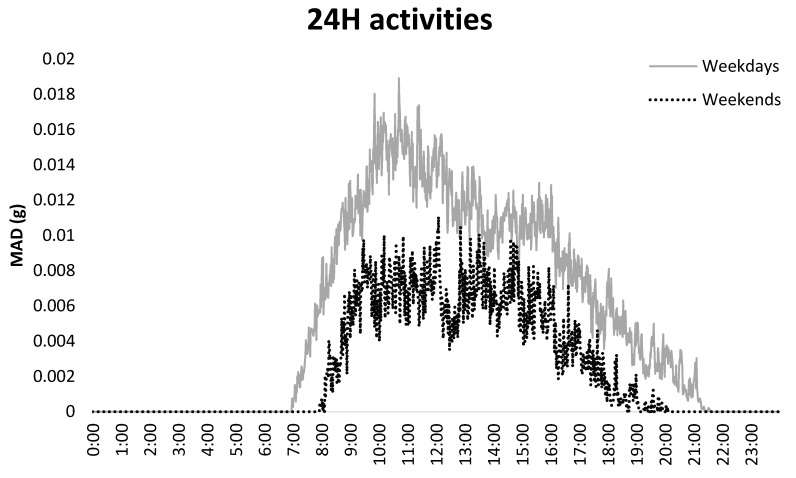
Accelerometer-assessed mean amplitude deviation (MAD) of 24 h of activities during weekdays and weekends. The median values are calculated from all participants.

During weekdays, participants had 1.6% less sedentary time (*p* < 0.001) and 1.5% more light activity (*p* < 0.001) than during weekends, and it was most notable in people without unmet PA need. People with unmet PA need had 2.1% less MVPA time (*p* = 0.005) compared to those without unmet PA need and it was apparent in both weekdays (*p* = 0.008) and weekends (*p* = 0.019) (Table 2). Figure 2 presents MAD results in each minute of 24 h of activities during weekdays and weekends. Furthermore, the averaged 24 h visualization was reported as a Supplementary Figure S1. 

## 4. Discussion

Little work has been done to explore the daily PA activity patterns and sedentary time accumulation within weekdays and weekend days, and to take account of unmet PA need as an indicator of willingness to engage in more PA among older people. We found that older people have slightly more light activity and less sedentary time during weekdays than during weekend days. Furthermore, the ones perceiving themselves not to have an adequate level of PA were found to spend less time in MVPA, especially during weekdays.

The mean daily sedentary time in our study was higher than has been reported in Finnish-representative samples for older adults [28]. Across all measured days, we found that older people spent more than 10 h per day being sedentary. In the sub-sample of the population-based Health 2011 Study of Finnish adults, the mean sedentary time of those aged 70–85 years was about 9 h per day, measured using the same accelerometer and MAD method as was used in the current study [28]. Husu and colleagues (2016) included a population representative sample of 70–85 year olds, whereas we preferentially sampled in the wide range of 75 to 90 [28]. This difference may be partly explained by aging being involved with more sedentary behavior and less mobility [29].

Our study provides evidence that weekdays and weekend days may need to be considered independently among older people in order to obtain the level of detail required to design efficacious intervention strategies. In agreement, Marshall and colleagues (2015) reported that although the total accelerometer-measured sedentary time did not differ between weekdays and weekend days, older adults may tend to have a behavioral compensation mechanism occurring across the week [20]. For example, some more active older adults who typically engage in less sedentary time on weekdays tended to be more sedentary during weekend days. Conversely, those older adults who engaged in more sedentary time on weekdays tended to be less sedentary during the weekend [20]. When we focused on the specific times of the day (Supplementary Figure S1), we observed differences in the timings of PA between a weekday and a weekend day. On the weekdays, the most activities appeared between 10:00 and 12:00, which corresponds to a typical lunch time in Finnish society. On the weekend days, the most active epoch appeared in the afternoon, from 15:00 to 16:00, which is likely to be associated with social routines, e.g., shopping or walking outside [22]. 

It seems that many factors may lead to unmet PA need in ambulatory community-living older people. For example, Rasinaho and colleagues reported that people who reported a perceived difficulty in walking were more willing to increase their PA than those who did not report a difficulty [30]. There is a negative association between less favorable environmental features in the neighborhood and participation in PA [31], especially among those with unmet PA need with impeded mobility [8]. We have previously suggested that unmet PA need could be a consequence of a discrepancy between environmental demands and reduced individual capabilities [8]. In the present study, we found that the older people without unmet PA need had almost twice the amount of MVPA (equal to 16 min/d of MVPA) compared to those with an unmet PA need. It seems that the people who perceived unmet PA need may in fact have declined activity and consequently have less MVPA during their daily lives, although they wish to engage more in MVPA. Those differences further appeared with more sedentary time and less time spent in light and MVPA in both weekdays and weekends, for people who perceived unmet PA need compared to those without unmet PA need. Previously we suggested that there is a linear positive relationship between PA and life-space mobility PA [32]. Thus, we suggest providing more PA possibilities and environmental supports for those older people who perceive unmet PA need in order to help them to maintain a higher life-space mobility [33]. 

In this study, we used raw accelerometry data from the sub-study of LISPE project [21]. Our previous publications reported PA results based on the manufactory output or self-reported questionnaires [34,35]. However, in the current study, we used the recently proposed the universal method of MAD for the commensurate assessment of raw accelerometer data [24] It provides enough sensitivity and specificity to compare it with common accelerometer brands and to validate the MAD-based cut-offs for sedentary, light, moderate and vigorous intensity activities against oxygen consumption [25,36]. 

The present study provides new information regarding the detailed PA differences at particular times of the day within a weekday and weekend day among older people. In addition, a novelty of this study was the consideration of unmet PA need as an indicator to provide evidence of whether the perceived adequacy of PA is related to the measured amount of PA and sedentary time. However, it should be noted that in the current study, only 13 participants perceived unmet PA need. The LISPE project is a population-based cohort study. However, as is often the case, those with major mobility difficulties or poor health were less willing to participate in a research study, especially when more effort and commitment are required [37]. Our sample of older people was comparatively small from the LISPE sub-study [21] and may have a decidedly higher PA level overall and be more active than other participants, as they were willing to wear accelerometers in this study. Thus, the findings cannot be generalized to the total population of older people. Moreover, it is worth noting that those with unmet PA need from the LISPE project were less likely to participate in a sub-study with accelerometer wearing compared to those without. Despite the low number of participants with unmet PA need, differences compared to those without were observed, which seems to indicate that unmet PA need is rather strongly linked with actual physical behavior, and could therefore help identify those who could potentially benefit from enabling interventions. Finally, while the protocol called for 7-day wear to ensure that both weekdays and weekend days were included in the analyses, we did not get a full 7-day sample from all participants. This is a typical issue in free-living accelerometry, and we applied a minimum of three successfully recorded days as the inclusion criterion [38].

## 5. Conclusions

Older people have slightly more light activity and less sedentary time during weekdays than during weekends, which may relate to opportunities for PA. Furthermore, those perceiving themselves not to have an adequate level of PA were found to spend less time in MVPA, especially during weekdays.

## Figures and Tables

**Table 1:** Participant characteristics, physical activity (PA), and sedentary time.

Mean ± SD	All (*n* = 174)
Age, years	79.9 ± 4.3
Female, % (*n*)	63.8 (111)
Unmet PA need *, % (*n*)	7.6 (13)
Accelerometer-derived variables	
No. days	6.3 ± 1.1
Removed days	0.6 ± 1.1
Recording time/day (h)	13.5 ± 1.3
Sedentary time (min/d)	607.8 ± 82.9
Light PA (min/d)	163 ± 61.9
MVPA (min/d)	37.9 ± 29.4
Sedentary bouts (>10 min/d)	16.6 ± 2.9
Light and MVPA bouts (>10 min/d)	0.8 ± 0.9

* missing *n* = 2.

**Table 2:** Accelerometer-derived variables for those with unmet PA need and without unmet PA need during weekdays and weekends.

	Daily		Weekdays		Weekends	
	With (*n* = 13)	Without (*n* = 159)	With (*n* = 13)	Without (*n* = 159)	With (*n* = 13)	Without (*n* = 159)
Recording time (h/d)	13.3 ± 0.8	13.5 ± 1.3	13.3 ± 1.0	**13.6 ± 1.4 #**	13.5 ± 1.0	**13.2 ± 1.5 #**
Sedentary time (%)	76.7 ± 8.9	75.1 ± 8.8	76.2 ± 8.7	**74.7 ± 9.0 #**	77.9 ± 9.7	**75.9 ± 9.5 #**
Light PA (%)	20.6 ± 7.8	20.0 ± 7.2	21.0 ± 7.6	**20.5 ± 7.4 #**	19.5 ± 8.8	**19.2 ± 8.1 #**
MVPA (%)	2.7 ± 3.4 *	4.8 ± 3.5 *	2.8 ± 3.4 *	4.9 ± 3.6 *	2.6 ± 3.6 *	4.6 ± 4.1 *
Sedentary bouts (>10 min/d)	16.9 ± 2.2	16.6 ± 2.9	16.7 ± 2.4	16.7 ± 3.1	17.2 ± 2.7	16.5 ± 3.6
Light and MVPA bouts (>10 min/d)	0.6 ± 1.1	0.9 ± 0.9	0.6 ± 1.1	0.8 ± 0.9	0.8 ± 1.4	0.9 ± 1.1

* Bold values indicate a significant difference between with vs. without unmet PA need (*p* < 0.05); # Bold values indicate a significant difference between weekdays vs. weekends (*p* < 0.05).

## Supplementary Materials

The following are available online at https://www.mdpi.com/1660-4601/17/18/6887/s1, Figure S1: Accelerometer-assessed mean amplitude deviation (MAD) of 24 h of activities during weekdays and weekends. The average values are calculated from all participants.
