# Supplementary material for: Daily Physical Activity and Sedentary Time Assessed by Acceleration Based on Mean Amplitude Deviation among Older People
Source: Int J Environ Res Public Health. 2020 Sep 21;17(18):6887. doi: 10.3390/ijerph17186887 (PMC7560095; doi:10.3390/ijerph17186887)
Supplement: Supplementary file 1 [file ijerph-17-06887-s001.pdf]

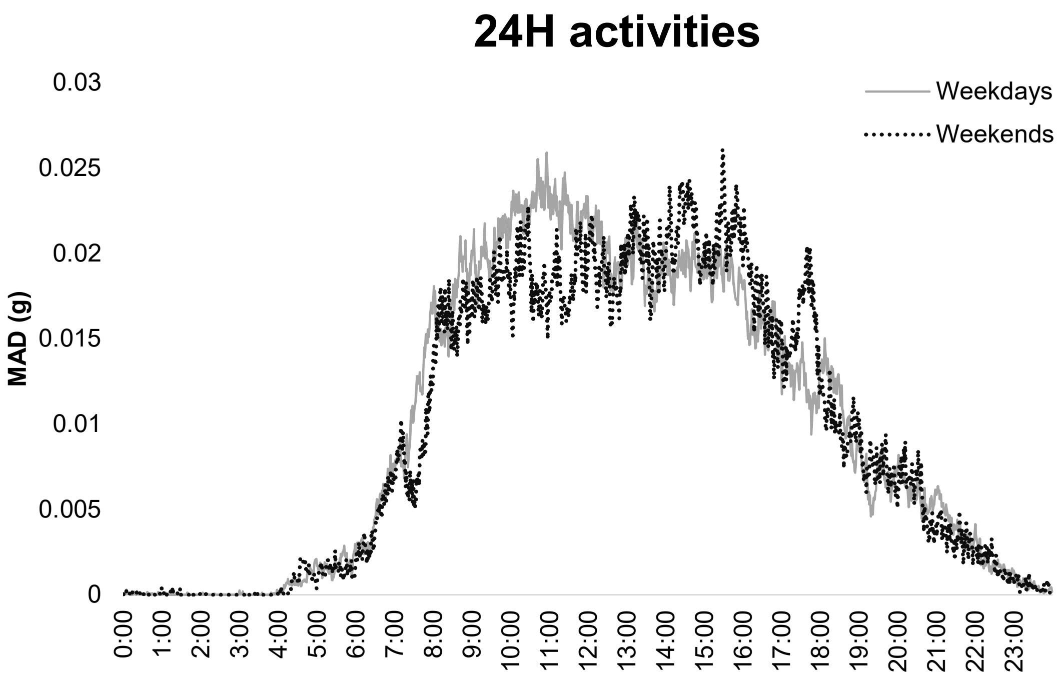

Supplementary Figure S1. Accelerometer-assessed mean amplitude deviation (MAD) of 24 h of activities during weekdays and weekends. The average values are calculated from all participants.
